# Mid-Pleistocene transition in glacial cycles explained by declining CO_2_ and regolith removal

**DOI:** 10.1126/sciadv.aav7337

**Published:** 2019-04-03

**Authors:** M. Willeit, A. Ganopolski, R. Calov, V. Brovkin

**Affiliations:** 1 Potsdam Institute for Climate Impact Research, Potsdam, Germany.; 2 Max Planck Institute for Meteorology, Hamburg, Germany.

## Abstract

Variations in Earth’s orbit pace the glacial-interglacial cycles of the Quaternary, but the mechanisms that transform regional and seasonal variations in solar insolation into glacial-interglacial cycles are still elusive. Here, we present transient simulations of coevolution of climate, ice sheets, and carbon cycle over the past 3 million years. We show that a gradual lowering of atmospheric CO_2_ and regolith removal are essential to reproduce the evolution of climate variability over the Quaternary. The long-term CO_2_ decrease leads to the initiation of Northern Hemisphere glaciation and an increase in the amplitude of glacial-interglacial variations, while the combined effect of CO_2_ decline and regolith removal controls the timing of the transition from a 41,000- to 100,000-year world. Our results suggest that the current CO_2_ concentration is unprecedented over the past 3 million years and that global temperature never exceeded the preindustrial value by more than 2°C during the Quaternary.

## Teaser

Model simulations reveal the importance of atmospheric CO_2_ and glacial erosion of regolith for Quaternary climate dynamics.

## INTRODUCTION

The Quaternary is characterized by the appearance of glacial-interglacial cycles caused by the cyclic growth and decay of continental ice sheets in the Northern Hemisphere (NH). Before the initiation of NH glaciation at ~2.7 million years (Ma) ago, as indicated by the appearance of ice-rafted debris in the North Atlantic, the growth of NH ice sheets was probably suppressed by elevated atmospheric CO_2_ (*1*, *2*). Afterward, benthic δ^18^O records (*3*) show a trend toward larger ice sheets and colder climate over the Quaternary, together with an increase in the amplitude of the glacial-interglacial variability (*4*). Of particular interest is the transition between ~1.25 and ~0.7 Ma ago, known as the mid-Pleistocene transition (MPT) (*5*–*7*), from mostly symmetric cycles with a period of about 41 thousand years (ka) to strongly asymmetric 100-ka cycles. Several hypotheses for the mechanism of the MPT have been proposed. One of them invokes a gradual decline of CO_2_ during the past 3 Ma to explain both the onset of Greenland (*2*, *8*) and, more generally, NH glaciations (*1*) and the MPT transition (*9*–*11*). Another hypothesis attributes the MPT to a gradual removal of a thick regolith layer from North America and northern Europe (*12*–*14*).

The atmospheric CO_2_ concentration is accurately known only for the past ~800 ka, the period covered by ice core data. Nevertheless, proxy-based reconstructions suggest that, over the past ~2 Ma, CO_2_ was not very different from the concentrations measured in ice cores (*15*–*17*) but that it was substantially higher during the late Pliocene (*18*, *19*).

It has been postulated that NH continents were all covered by regolith before the Quaternary, an expected outcome of the 10^7^ to 10^8^ years that the bedrock was exposed to weathering before the initiation of glacial cycles (*7*). The observed present-day regolith distribution (*20*, *21*), which is characterized by exposed bedrock over large parts of northern North America and Eurasia, is a result of glacial erosion by Quaternary ice sheets. A gradual removal of regolith by glacial erosion could have changed the ice sheets’ response to orbital forcing. Changes in regolith distribution may affect glacial cycles through several mechanisms. The first one is based on the fact that, in the case of temperate-base ice sheets, the sliding velocity of ice is much higher in the presence of a thick regolith layer as compared to exposed rocks (*12*). This makes ice sheets more mobile, thinner, and more susceptible to orbital forcing. In addition to this mechanism, Ganopolski and Calov (*13*) also found that, in the case when ice sheets expand well into areas covered by regolith, enhanced deposition of glaciogenic dust over the southern margins of NH ice sheets substantially lowers snow albedo, thereby facilitating melting and preventing growing of large ice sheets.

It has been shown that modeling of long (100 ka) and strongly asymmetric glacial cycles of the late Quaternary requires both the presence of large areas of northern continents with exposed rocks and a relatively low atmospheric CO_2_ concentration (*13*, *22*, *23*). Here, we investigate the origin of the major transitions in Quaternary climate dynamics by performing a large set of transient simulations with the Earth system model of intermediate complexity CLIMBER-2 (*24*).

## RESULTS

### Transient model simulations

CLIMBER-2 includes atmosphere, ocean, vegetation, global carbon, and dust models and the three-dimensional thermomechanical ice sheet model SICOPOLIS (*25*). It has been recently applied for simulating the last four glacial cycles with a fully interactive carbon cycle (*26*). There, we demonstrated that glacial lowering of atmospheric CO_2_ in the model is controlled by lowered sea surface temperatures (SSTs) and changes in ocean circulation, in particular enhancement of Antarctic bottom waters and decrease of deep ocean ventilation. Elevated carbonate weathering on exposed shelves and enhanced nutrient utilization in the Southern Ocean due to enhanced dust deposition also play important roles, especially toward glacial maxima (*27*). Reorganizations of the Atlantic meridional overturning circulation during glacial terminations contribute substantially to deglacial CO_2_ rise. The terrestrial carbon cycle, which includes novel components such as permafrost carbon, peat, and carbon buried under ice sheets, plays a minor role in atmospheric CO_2_ dynamics on orbital time scales (*26*).

To perform multiple simulations covering the entire Quaternary, we use a novel technique of splitting long-term simulations over the past 3 Ma into shorter intervals. In total, we perform more than 1000 model simulations, each 500 ka long, starting from the same initial conditions but at different points in time (Materials and Methods and fig. S1). This approach allows us to analyze the robustness of glacial cycles by testing whether different simulations converge to the same solution, and also has the advantage that it reduces the potential problem with long-term model drifts in the global carbon cycle. Model drifts can occur because, in our modeling setup, the carbon cycle is not closed. There are geologic sinks (burial in ocean sediments) and sources (carbonate weathering and volcanic CO_2_ outgassing), and even a tiny imbalance between them can cause a pronounced drift toward too high or too low CO_2_ concentrations on time scales of millions of years.

### CO_2_ outgassing and regolith scenarios

To test the effect of a gradual CO_2_ decrease on Quaternary climate dynamics, we use prescribed volcanic CO_2_ outgassing to control the mean CO_2_ concentration in the model. Small changes in volcanic outgassing represent a possible candidate to explain a long-term CO_2_ decrease, because even the value for the current volcanic outgassing is very uncertain (*28*). Alternatively, the same trend in CO_2_ can be explained by a similarly small increase in average weathering rate and/or organic carbon burial in deep-sea sediments (*29*).

**Fig. 1:**
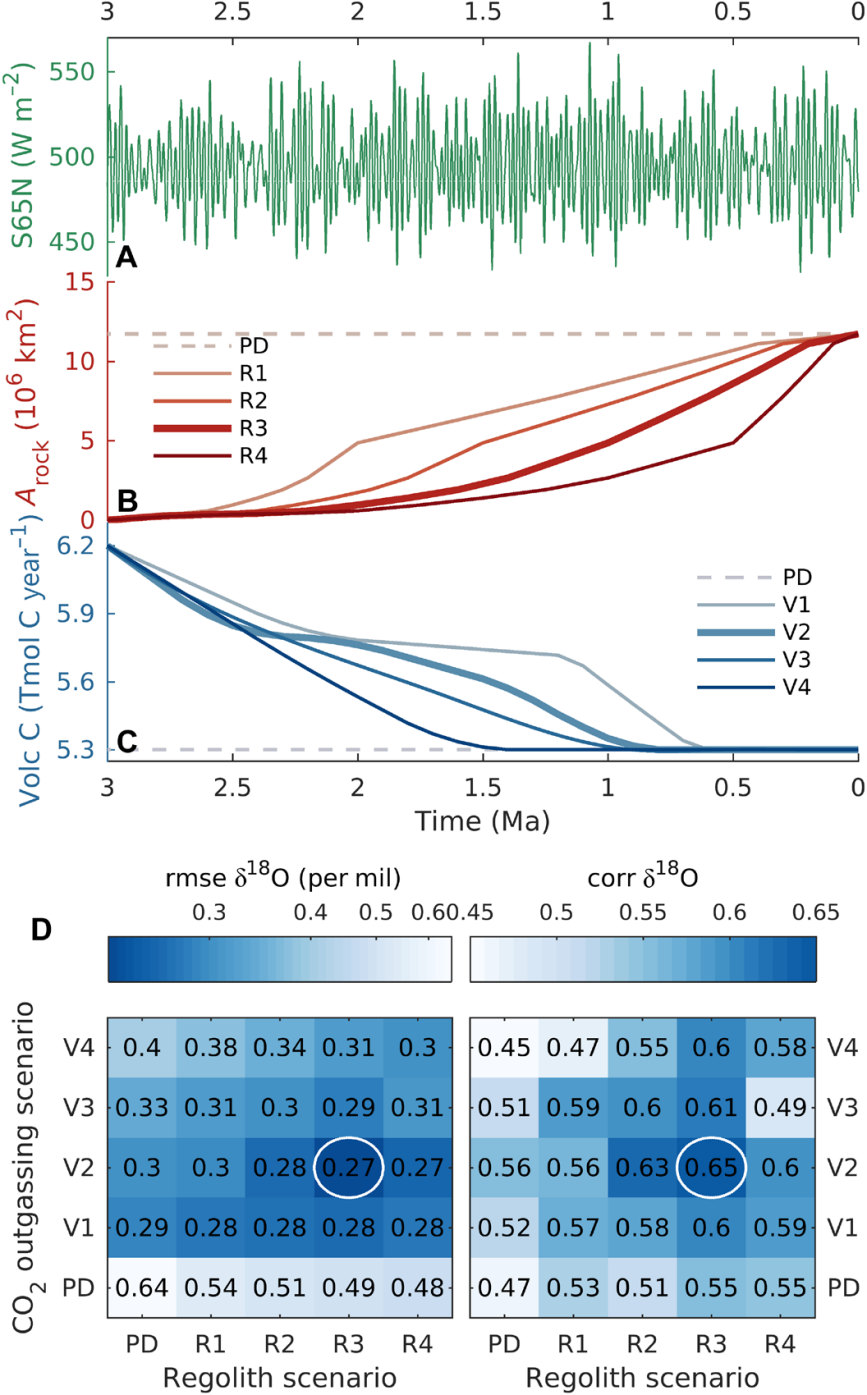
External drivers and best scenarios. (**A**) Summer solstice insolation at 65°N (S65N) (*44*). (**B**) Scenarios for the evolution of area of exposed crystalline bedrock (*A*_rock_) resulting from the gradual removal of regolith by glacial erosion over North America and Scandinavia (see Materials and Methods and fig. S3 for a spatially explicit illustration). (**C**) Volcanic CO_2_ (Volc C) outgassing scenarios (see Materials and Methods). (**D**) Root mean square error (rmse) and correlation (corr) between modeled and observed (*3*) benthic δ^18^O for simulations driven by the combination of different regolith removal and volcanic CO_2_ outgassing scenarios in (B) and (C). PD, present-day constant regolith and volcanic CO_2_ outgassing. The optimal scenarios, those minimizing root mean square error and maximizing correlation, are indicated by the white circles in (D) and by the thicker lines in (B) and (C).

On the basis of the evidence for CO_2_ decrease and regolith removal over the Quaternary, we created different scenarios for CO_2_ outgassing and regolith distribution, which were then used to drive the model together with orbital variations (see Materials and Methods; Fig. 1, B and C; and fig. S3). The need for these scenarios originates from the absence of appropriate models that can be used to simulate the evolution of these two factors on the million-year time scale. The initial and final values of volcanic outgassing are determined using an inverse modeling approach (see Materials and Methods), while the initial and final spatial distributions of regolith are either known from observation or strongly constrained by empirical data. Therefore, the scenarios differ only in their temporal evolution (Fig. 1, B and C). For each of the 16 different combinations of regolith and volcanic outgassing scenarios, we run the model over the past 3 Ma using the time-splitting technique and selected the best scenario by minimizing the difference between simulated and observed benthic δ^18^O (Fig. 1D; see also Materials and Methods) (*3*).

### Transient simulations with optimal CO_2_ outgassing and regolith scenarios

**Fig. 2:**
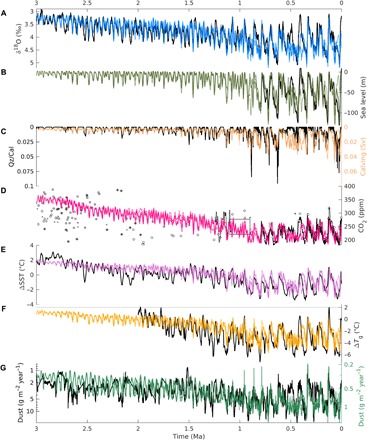
Transient modeling results. Results of model simulations driven by orbital forcing, optimal regolith removal scenario, and optimal volcanic outgassing scenario. In all panels, observations are shown in black and model results are shown as colored lines. (**A**) Benthic δ^18^O compared to the stack of (*3*). (**B**) Relative sea level compared to (*49*). (**C**) Calving from the Laurentide ice sheet into the North Atlantic compared to a proxy for ice-rafted debris at site U1313 (*30*). (**D**) Atmospheric CO_2_ concentration compared to ice core data (solid line) (*50*) and other proxies [circles: (*16*); squares: (*18*); *: (*51*); + and ×: (*19*); diamonds: (*52*); black box: (*15*); dotted lines: (*17*)]. (**E**) SST anomalies compared to the stack of (*18*). (**F**) Global annual surface air temperature compared to reconstructions (*53*). (**G**) Southern Ocean dust deposition compared to data (*54*).

**Fig. 3:**
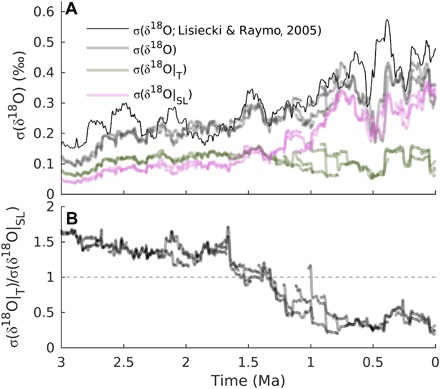
Benthic δ^18^O decomposition. (**A**) Contribution of sea level and deep ocean temperature to benthic δ^18^O variability through time, computed as moving SD with a window of 150 ka. The modeled total δ^18^O variability (gray lines) is compared to the variability of the stack from (*3*) (black). The modeled contribution of deep ocean temperature and sea level to δ^18^O variability is shown by the green and magenta lines, respectively. The decomposition in terms of sea level, *z*_SL_, and deep ocean temperature, *T*_d_, is derived using the following formula: δ^18^O = 4.0 − 0.22 *T*_d_ − 0.01 *z*_SL_. (**B**) Ratio between deep ocean temperature and sea-level contribution to the total δ^18^O variability.

**Fig. 4:**
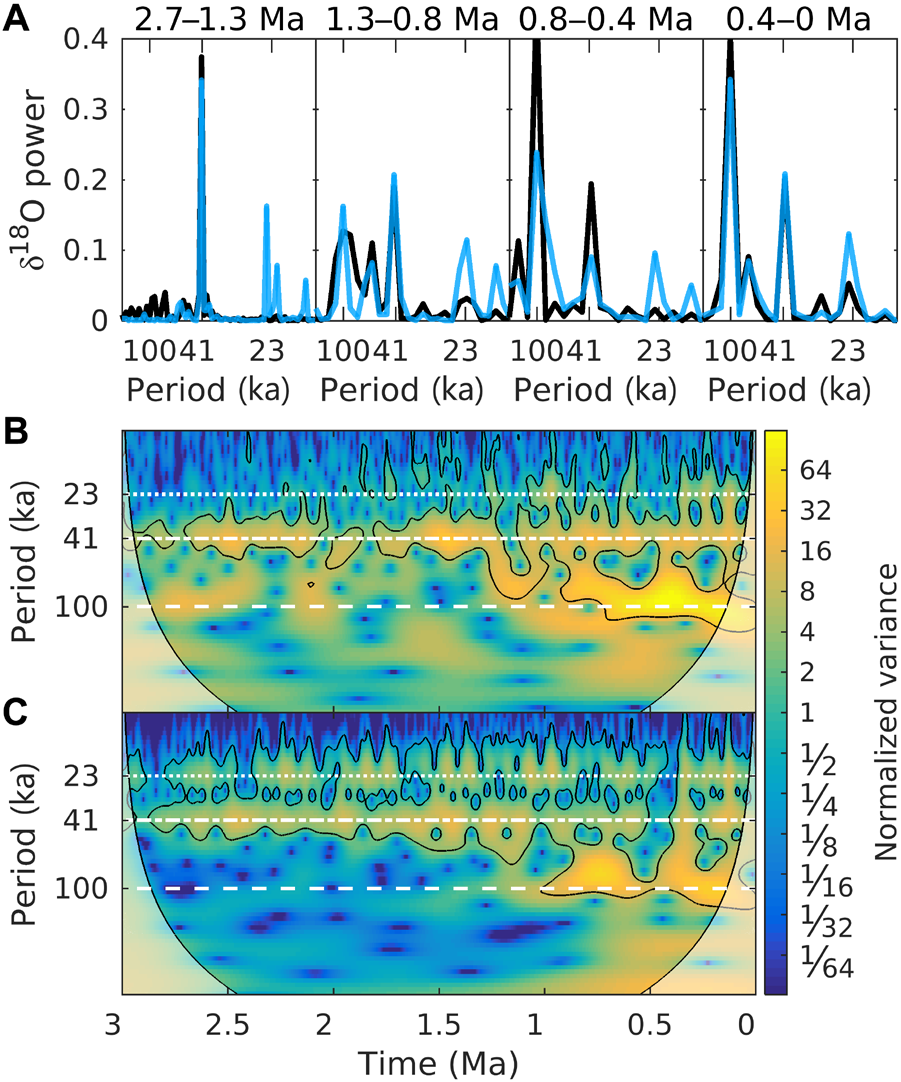
Power and wavelet spectra of benthic δ^18^O. (**A**) Power spectra of modeled δ^18^O (blue) compared to power spectra of the δ^18^O stack of (*3*) (black) for different time intervals, as indicated above the panels. Wavelet spectra of (**B**) the benthic δ^18^O stack of (*3*) and (**C**) modeled δ^18^O. Black contours indicate the 5% significance level against red noise. The horizontal white lines represent the orbital periods of precession (∼23 ka), obliquity (∼41 ka), and eccentricity (∼100 ka). The model spectra are derived from the average δ^18^O computed over all ensemble members resulting from the time-splitting technique.

**Fig. 5:**
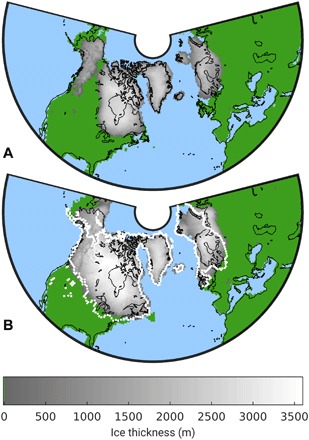
Pre- and post-MPT ice sheets. Modeled maximum ice thickness in each grid cell (**A**) before and (**B**) after the MPT. The dotted lines in (B) indicate the reconstructed ice extent at the last glacial maximum.

When the model is driven by orbital variations and the optimal regolith and volcanic outgassing scenarios, it reproduces the evolution of many reconstructed characteristics of Quaternary glacial cycles (Fig. 2). It simulates most of the details of the observed benthic δ^18^O curve (Fig. 2A), including long-term trends and glacial-interglacial variability. The relative contribution of deep-sea temperature and sea-level variations to δ^18^O variability changes substantially through time, with temperature variations being more important during the early Quaternary and sea-level variations dominating the signal during the late Quaternary (Fig. 3). The model also captures the secular cooling trend of ~−1°C/Ma in SSTs (Fig. 2E). The intensification of NH glaciation after ~2.7 Ma ago is marked by a rather abrupt increase in global ice volume variations (Fig. 2B) and an increase in ice flux from the Laurentide ice sheet into the North Atlantic, in good agreement with a proxy for ice-rafted debris (Fig. 2C) (*30*). Interglacial atmospheric CO_2_ concentrations decrease from values of ~350 parts per million (ppm) during the late Pliocene to values between 260 and 290 ppm, typical of the past 800 ka, at ~1 Ma ago (Fig. 2D). The amplitude of glacial-interglacial CO_2_ variations increases from ~50 ppm at the beginning of the Quaternary to ~80 to 90 ppm during the 100-ka cycles of the past million years. This suggests that, for the early Quaternary, the large spreading between and within different CO_2_ reconstructions markedly overestimates real CO_2_ variability. In agreement with (*17*), a substantial fraction of the increase in the magnitude of glacial-interglacial CO_2_ changes is attributed to a larger contribution of the iron fertilization mechanism, which, in turn, is related to an increase in dust deposition rate over the Southern Ocean during late Quaternary glacial cycles (Fig. 2G). Various previous modeling studies have attempted to derive continuous CO_2_ records for the pre–ice core time using different methods and assumptions. van de Wal *et al.* (*31*) derived CO_2_ concentrations that are substantially lower than our estimates for the late Pliocene and early Quaternary, with values never exceeding 300 ppm (fig. S6B). The more recent reconstruction by (*32*) shows a much larger glacial interglacial variability, particularly before the MPT, compared to our results. The CO_2_ scenario that we derived for the Pliocene-Pleistocene between 3 and 2.4 Ma ago in a previous study (*1*) is comparable to the results presented in this study (fig. S6B). During the late Pliocene, simulated global surface air temperature varies by less than 1°C in response to variations in Earth’s orbit, with interglacial temperatures ~1.5°C warmer than at preindustrial (Fig. 2F). Successively, glacial-interglacial temperature variability gradually increases, reaching values up to ~6°C during the past million years. Simulated global temperatures never exceed 2°C above the preindustrial over the past 3 Ma (Fig. 2F). At ~1 Ma ago, the main periodicity of modeled δ^18^O variations changes from 41 to 100 ka (Fig. 4). Before that, the model mostly responds to obliquity forcing at the 41-ka period, in agreement with data (Fig. 4). However, the model shows a larger response to precession than is observed, possibly because of a missing dynamic Antarctic ice sheet in the model (*33*) or because the benthic δ^18^O stack is orbitally tuned to obliquity (*3*). SST, CO_2_, and sea level show a similar transition from 41 to 100 ka (fig. S5). The ice sheets are generally thinner before compared to after the MPT (Fig. 5), but the pre- and post-MPT maximum areal ice extent is comparable over Scandinavia and eastern North America, broadly in accordance with available observations (*7*). The model results are compared to additional observations in fig. S6.

The fact that our model has problems at simulating the strong glacial cycle between 500 and 400 ka ago is not surprising because it occurred during a period of very weak orbital forcing. There were so far only few attempts to simulate this period of time with sufficiently realistic climate–ice sheet models (*9*, *34*), and they all reveal problems with simulation of this time interval, suggesting that current climate–ice sheet models are not nonlinear enough to properly simulate this challenging interval.

### Robustness of glacial cycles

The question of whether glacial cycles are deterministic or stochastic has been highly debated, and a complete spectrum of opinions—from chaotic to perfectly deterministic—exists (*35*–*37*). In most cases, our model simulations that started at different points in time converge to one and the same solution (Fig. 2 and fig. S1), close to reconstructions, providing a strong indication that glacial cycles are robust. The glacial cycles of the 41-ka world are particularly robust, which is expected because they represent a relatively linear response to variations in obliquity (*4*). The 100-ka cycles appear to be less robust, because they involve a much more nonlinear response to orbital forcing. However, the good agreement between the bulk of the model simulations and the reconstructed glacial cycles evolution over the past 1 Ma (Fig. 2A), except for MIS11, indicates that the observed realization was the most likely, although not the only possible one. This finding is fully consistent with that of Tzedakis *et al*. (*35*), who demonstrated that the most probable timing of the most recent glacial terminations is close to that seen in paleoclimate records.

### Separate effects of CO_2_ outgassing and regolith scenarios

**Fig. 6:**
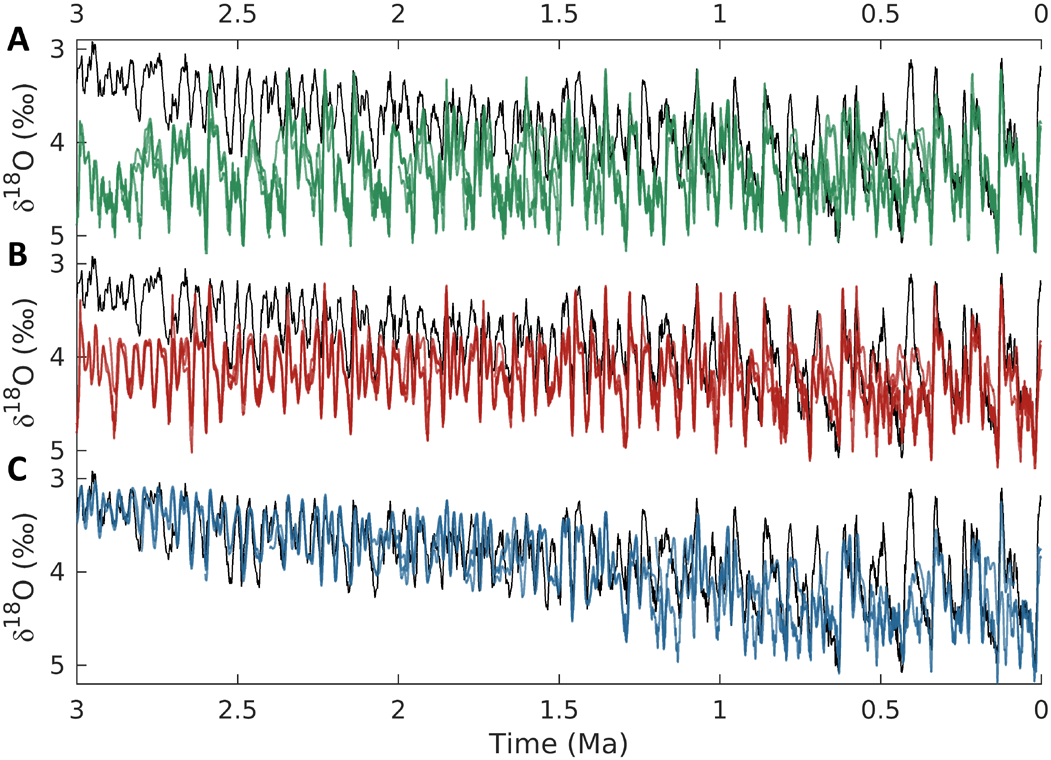
External drivers and model response. Modeled benthic δ^18^O compared to observations (black) (*3*) for (**A**) simulations driven only by variations in orbital configuration (green), (**B**) simulations driven by orbital forcing and optimal regolith removal scenario (red), and (**C**) simulations driven by orbital forcing and optimal volcanic outgassing scenario (blue).

To assess the influence of the different factors on Quaternary glacial cycles, we performed a set of additional experiments where we fixed regolith distribution and/or volcanic outgassing to present-day conditions. When the model is driven by orbital variations as the only external forcing (Fig. 1A), modeling results and data diverge markedly before the MPT, with the model simulating pronounced 100-ka cycles throughout the whole 3 Ma (Fig. 6A). These results confirm that orbital forcing alone cannot explain the evolution in Quaternary glacial cycles. When the optimal regolith removal scenario (Fig. 1B) is prescribed additionally to orbital forcing, but volcanic outgassing is held constant, the model simulates a transition from 41- to 100-ka cycles (Fig. 6B), with the precise timing of this transition depending on the regolith removal scenario (fig. S4). At the same time, these simulations fail to reproduce the general increase in δ^18^O between 3 and 1 Ma ago (Fig. 6B) and the reconstructed global cooling trend in SSTs (fig. S8D). Last, a decrease in volcanic CO_2_ outgassing (Fig. 1C), together with orbital forcing but with the prescribed present-day regolith cover, captures the trends in δ^18^O (Fig. 6C) and SSTs (fig. S2C). It can also explain a transition from 41- to 100-ka glacial cycles, but for any plausible CO_2_ scenario, this transition occurs much earlier than in reality (fig. S2).

## DISCUSSION

Our transient modeling results demonstrate that both previously proposed mechanisms—regolith removal and gradual lowering of CO_2_—are essential to reproduce the realistic evolution of climate variability during the Quaternary, and their combination controls the timing of regime changes of climate variability. Note that a gradual change of the regolith cover causes a rather rapid (few hundred thousand years) transition from the 41- to 100-ka world, in good agreement with observational data. Simulated glacial cycles only weakly depend on initial conditions and therefore represent a quasi-deterministic response of the Earth system to orbital forcing. Our results also support the notion that the current CO_2_ concentration of more than 400 ppm is unprecedented over at least the past 3 Ma and that global temperature did not exceed the preindustrial value by more than 2°C during the Quaternary. In the context of future climate change, this implies that a failure in substantially reducing CO_2_ emissions to comply with the Paris Agreement target of limiting global warming well below 2°C will not only bring Earth’s climate away from Holocene-like conditions but also push it beyond climatic conditions experienced during the entire current geological period.

The results of our study are based on an Earth system model of intermediate complexity, whose high computational efficiency needed for simulations on a million-year time scale is achieved by using a rather coarse spatial resolution and considerable simplifications in the description of individual processes, in particular atmospheric dynamics. Further progress in understanding of Quaternary climate dynamics would require the use of complex Earth system models. However, moving Quaternary modeling to a qualitatively new level would require not only the use of existing complex models but also substantial progress in modeling of ice sheet–solid Earth interaction (*38*) and, in particular, its impact on long-term landscape evolution, sediments transport (*21*), global dust and carbon cycles, and other processes that are not yet properly understood.

## MATERIALS AND METHODS

### Model

For this study, we used the Earth system model of intermediate complexity CLIMBER-2 (*24*), which incorporates the three-dimensional thermomechanical ice sheet model SICOPOLIS (*25*). SICOPOLIS is a shallow ice approximation model, which treats marine ice by allowing grounded ice to propagate over the continental shelf. This enables the model to resemble to a good approximation the Earth ice cover during the Quaternary, including grounded marine ice, which is a considerable portion of the northern European ice cover during cold phases. SICOPOLIS is applied only to the NH with a spatial resolution of 1.5° × 0.75° and is fully interactively coupled to the low-resolution climate component and a model of deep permafrost (*39*). Meltwater and iceberg fluxes directly affect ocean circulation. Forced by orbital variations and prescribed radiative forcing from greenhouse gases, the model has been applied to simulate the last eight glacial cycles (*13*). CLIMBER-2 also includes a global carbon cycle model (*27*) and has been the first model to reproduce the main characteristics of the last four glacial cycles with orbital forcing as the only prescribed external forcing (*26*). Because CLIMBER-2 does not include methane and nitrous oxide cycles and does not account for these greenhouse gases in its radiative scheme, we made use of the fact that CO_2_ is the dominant greenhouse gas and that, on orbital time scales, variations of the other two follow rather closely CO_2_ during the past 800 ka (*40*). To account for the effect of methane and nitrous oxide on radiative forcing, we computed the effective CO_2_ concentration used in the radiative scheme of the model in such a way that radiative forcing of equivalent CO_2_ exceeds radiative forcing of simulated CO_2_ by 30% at any time (*26*). Unlike the previous model version, the model used in this study also includes a fully interactive dust cycle model (*41*), with the atmospheric dust load directly affecting the shortwave radiative balance of the atmosphere and dust deposition on snow reducing surface albedo. Benthic δ^18^O is estimated from the modeled sea level, *z*_SL_, and deep ocean temperature, *T*_d_, as follows: δ^18^O = 4.0 − 0.22 *T*_d_ − 0.01 *z*_SL_. The temperature sensitivity factor is from (*42*), and the sea-level factor is from (*43*). Sea level is computed from the volume of modeled NH ice sheets assuming an additional 10% contribution from Antarctica. It has to be noted that this assumption rules out possible canceling NH/Southern Hemisphere precessional-phase contributions for δ^18^O and sea level (*33*).

The SST used to compare the stack of (*18*) is computed as the annual average SST of the model grid cells that contain the sediment cores from which the stack of (*18*) was derived. The SST computed this way turns out to differ substantially from the global SST in the model, particularly in the long-term trend.

### Transient simulations, splitting in time technique

We then used CLIMBER-2 to perform transient simulations of the past 3 Ma. In principle, it would be possible to perform one single transient simulation from 3 Ma ago to the present, but this would be impractical because of the long time (more than a month) it would take for a complete simulation. In addition, even very small imbalances in the carbon cycle would cause the model to drift away on such long time scales. We therefore applied a splitting in time technique, which consists of running many model simulations for 500 ka starting at different times from 3.25 Ma ago until 150 ka ago at time intervals of 100 ka. For all runs, the model was initialized using identical preindustrial interglacial conditions as described in (*26*). All model runs were thus started from the same initial state of the climate-carbon cycle–ice sheet system, but with differing orbital configuration, regolith distribution, and volcanic CO_2_ outgassing according to the initial astronomical time. We then discarded the initial 100 ka of each simulation as model spin-up and analyzed the remaining 400 ka (fig. S1).

The application of the time-splitting method generates an ensemble of model simulations that are (partly) overlapping in time and may not necessary converge to the same solution at all points in time. However, for spectral and wavelet analysis, a continuous time series is required. The simplest way to derive such a time series is by computing the mean over the ensemble members at each point in time. This is what is used in, e.g., Fig. 4. Using the ensemble median instead results in only small differences compared to the mean. The choice of the method to aggregate the ensemble simulations into one time series has therefore a negligible impact on the power and wavelet spectra.

### External forcings

In the transient simulations, we prescribed the changes in Earth’s orbital parameters, a schematic temporal evolution of regolith mask, and a time-dependent rate of volcanic CO_2_ outgassing as the only external drivers.

### Orbital forcing

Earth’s orbital parameters are well known for the past 3 Ma based on the astronomical solutions of (*44*).

### Regolith cover scenarios

The presence of regolith in our model has a dual effect on the ice sheets: (i) It enhances the velocity of ice sheet sliding over regions where the ice base is at its pressure melting point by a factor of 5, implying that basal velocity is five times higher for the same basal shear stress than for a sediment-free surface, roughly consistent with similar modeling approaches (*22*), and (ii) it increases the production of glaciogenic dust around the margins of the ice sheets, which affects surface albedo and facilitates surface melt (*45*). The present-day regolith mask was derived from the sediment thickness dataset of (*20*). Areas with sediment thickness larger than 100 m are assumed to be covered by regolith. The remaining areas, mainly large parts of North America and Scandinavia (fig. S3), are characterized by exposed crystalline bedrock. We assumed that the presently exposed bedrock areas are a result of glacial erosion by the Pleistocene ice flow associated with the waxing and waning of NH ice sheets and therefore considered all continents to be covered by regolith at 3 Ma ago, before the onset of NH glaciation. We then created 14 intermediate regolith masks by applying a two-dimensional backward diffusion process to the present-day regolith mask (fig. S3). Over Scandinavia, the applied diffusion coefficient is spatially uniform, while over North America, we started by diffusing the regolith mask northward from the southern margin of exposed bedrock until all continent is covered by regolith. In the resulting scenario, sediments were therefore first removed from the Arctic Archipelago, the area that we expect was the first to be affected by ice sheet growth at the onset of NH glaciation. Then, regolith was gradually removed in the area around the Hudson Bay and successively also further south and over Scandinavia. Some exposed bedrock is assumed to be present over Greenland at 3 Ma ago, but this assumption does not affect the results presented in the paper. With this procedure, we obtained the 16 regolith masks shown in fig. S3. By shifting these masks in time, we then created four scenarios that differ in the timing of regolith removal (Fig. 1B).

The most recent reconstruction of pre-Quaternary geography (*46*) indicates that the elevation was similar to present (no notable uplift during the past 3 Ma), but Hudson Bay and Canadian Arctic Archipelago were absent. Indirect data also suggest that the Hudson Bay only developed during the late Quaternary. These differences in geography do have some, but not critically important, impact on glacial cycles. However, the temporal evolution of geography is unknown, and we decided to prescribe present-day geography and elevation.

### Volcanic CO_2_ outgassing scenarios

To control the mean atmospheric concentration of CO_2_ in the model, we used small changes in the prescribed volcanic CO_2_ outgassing. The present-day volcanic outgassing used in the model is 5.3 Tmol C/year, which is the value that balances the weathering rate (*26*). The value of volcanic CO_2_ outgassing for ~3 Ma ago has been derived from fitting modeling results to the benthic δ^18^O stack. For that, we used an ensemble of transient model simulations initialized at 3.2 Ma ago and run for 500 ka driven by orbital variations, with continents fully covered by regolith and prescribed constant volcanic CO_2_ outgassing ranging from 5.3 to 6.5 Tmol C/year. The different values of volcanic CO_2_ outgassing lead to different modeled CO_2_ concentrations. A best fit of modeled benthic δ^18^O to the stack of (*3*) during interglacials is used to constrain the value of CO_2_ outgassing to 6.2 Tmol C/year for the time interval between 3 and 2.7 Ma ago. Some recent studies suggest that Antarctica could have contributed up to 20 m of sea-level equivalent during the late Pliocene/early Pleistocene (*47*, *48*). That would translate in a ~0.2‰ reduction in δ^18^O, which, in turn, would lead to lower volcanic CO_2_ outgassing values ~6.0 Tmol C/year to be more appropriate for the early Pleistocene. As a consequence, modeled atmospheric CO_2_ concentration would also be lower by ~30 ppm. We then constructed four different scenarios for the temporal evolution of volcanic CO_2_ outgassing between 3 Ma ago and the present day. All scenarios consist of a long-term decrease from 6.2 Tmol C/year at 3 Ma ago to 5.3 Tmol C/year at present, but the timing and rate of the decrease vary between scenarios (Fig. 1C). A decrease from 6.2 to 5.3 Tmol C/year corresponds to a decrease of less than 20%. Because even the value for the present-day volcanic outgassing is known with an uncertainty of more than 100% (~3 to 10 Tmol C/year) (*28*), changes in volcanic outgassing represent a possible candidate to explain the long-term CO_2_ decrease. However, the aim of our study is not to explain the causes of the CO_2_ decrease, and other processes such as changes in weathering (*7*) or organic carbon burial in deep-sea sediments (*29*) would also be potential candidates to explain the long-term CO_2_ decline.

### Optimal scenarios

Using the splitting in time technique described above, we then performed transient model simulations driven by orbital forcing and each of the 16 possible combinations of regolith removal and volcanic CO_2_ outgassing scenarios. We then selected the scenarios that gave the best fit, in terms of root mean square error and correlation, between modeled and observed δ^18^O (Fig. 1D). This is equivalent to solving an inverse problem to derive plausible scenarios for the poorly constrained long-term CO_2_ decrease and timing of regolith removal.

## Supplementary Material

http://advances.sciencemag.org/cgi/content/full/5/4/eaav7337/DC1

Download PDF

## SUPPLEMENTARY MATERIALS

Supplementary material for this article is available at http://advances.sciencemag.org/cgi/content/full/5/4/eaav7337/DC1 Fig. S1. Time-splitting technique. Fig. S2. Volcanic CO_2_ outgassing scenarios. Fig. S3. Regolith removal scenario. Fig. S4. Regolith scenarios. Fig. S5. Power spectra. Fig. S6. Comparison to additional observations and previous modeling results. Fig. S7. Transient simulations with present-day regolith and CO_2_ outgassing. Fig. S8. Transient simulations with present-day CO_2_ outgassing. Fig. S9. Transient simulations with present-day regolith. References (*55*–*58*)
