# Supplementary material for: Mid-Pleistocene transition in glacial cycles explained by declining CO2 and regolith removal
Source: Sci Adv. 2019 Apr 3;5(4):eaav7337. doi: 10.1126/sciadv.aav7337 (PMC6447376; doi:10.1126/sciadv.aav7337)
Supplement: http://advances.sciencemag.org/cgi/content/full/5/4/eaav7337/DC1 [file supp_5_4_eaav7337__index.html]

Science Advances | Science Advances

## Supplementary Materials

**This PDF file includes:**

- Fig. S1. Time-splitting technique.
- Fig. S2. Volcanic CO2 outgassing scenarios.
- Fig. S3. Regolith removal scenario.
- Fig. S4. Regolith scenarios.
- Fig. S5. Power spectra.
- Fig. S6. Comparison to additional observations and previous modeling results.
- Fig. S7. Transient simulations with present-day regolith and CO2 outgassing.
- Fig. S8. Transient simulations with present-day CO2 outgassing.
- Fig. S9. Transient simulations with present-day regolith.
- References (*55*–*58*)

Download PDF

**Files in this Data Supplement:**

- Adobe PDF - aav7337\_SM.pdf
