# Supplementary material for: Mid-Pleistocene transition in glacial cycles explained by declining CO2 and regolith removal
Source: Sci Adv. 2019 Apr 3;5(4):eaav7337. doi: 10.1126/sciadv.aav7337 (PMC6447376; doi:10.1126/sciadv.aav7337)
Supplement: Download PDF [file aav7337_SM.pdf]

Science Advances
AAAS

advances.sciencemag.org/cgi/content/full/5/4/eaav7337/DC1

Supplementary Materials for

Mid-Pleistocene transition in glacial cycles explained by declining CO_2 and regolith removal

M. Willeit*, A. Ganopolski, R. Calov, V. Brovkin

*Corresponding author. Email: willeit@pik-potsdam.de

Published 3 April 2019, *Sci. Adv.* **5**, eaav7337 (2019)
DOI: 10.1126/sciadv.aav7337

This PDF file includes:

Fig. S1. Time-splitting technique.
Fig. S2. Volcanic CO_2 outgassing scenarios.
Fig. S3. Regolith removal scenario.
Fig. S4. Regolith scenarios.
Fig. S5. Power spectra.
Fig. S6. Comparison to additional observations and previous modeling results.
Fig. S7. Transient simulations with present-day regolith and CO_2 outgassing.
Fig. S8. Transient simulations with present-day CO_2 outgassing.
Fig. S9. Transient simulations with present-day regolith.
References (*55–58*)

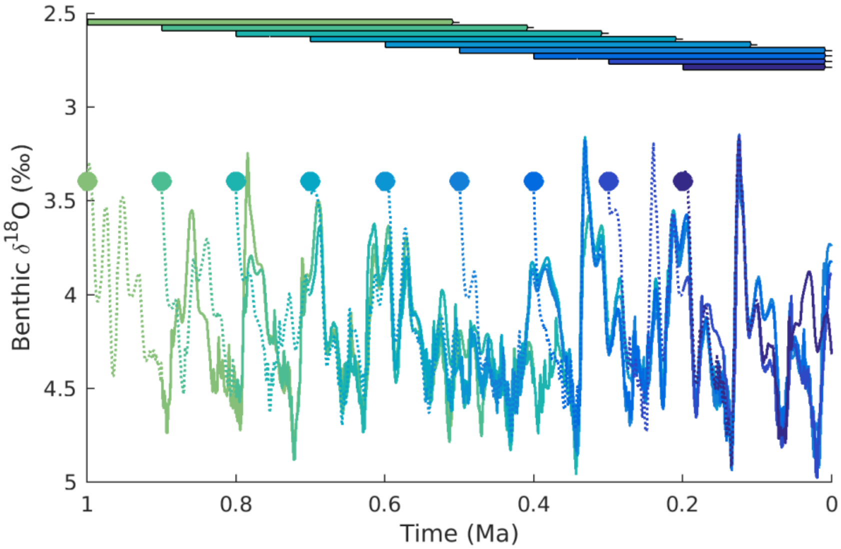

Fig. S1. Time-splitting technique. Illustration of the splitting in time technique for the last 1 Ma. Each model simulation initialized at different points in time, every 100,000 years, is 500,000 years long and is shown by a different color. The filled circles represent the initial points of the simulations. The first 100,000 years of each simulation, which are considered to be model spinup, are shown by the dotted lines.

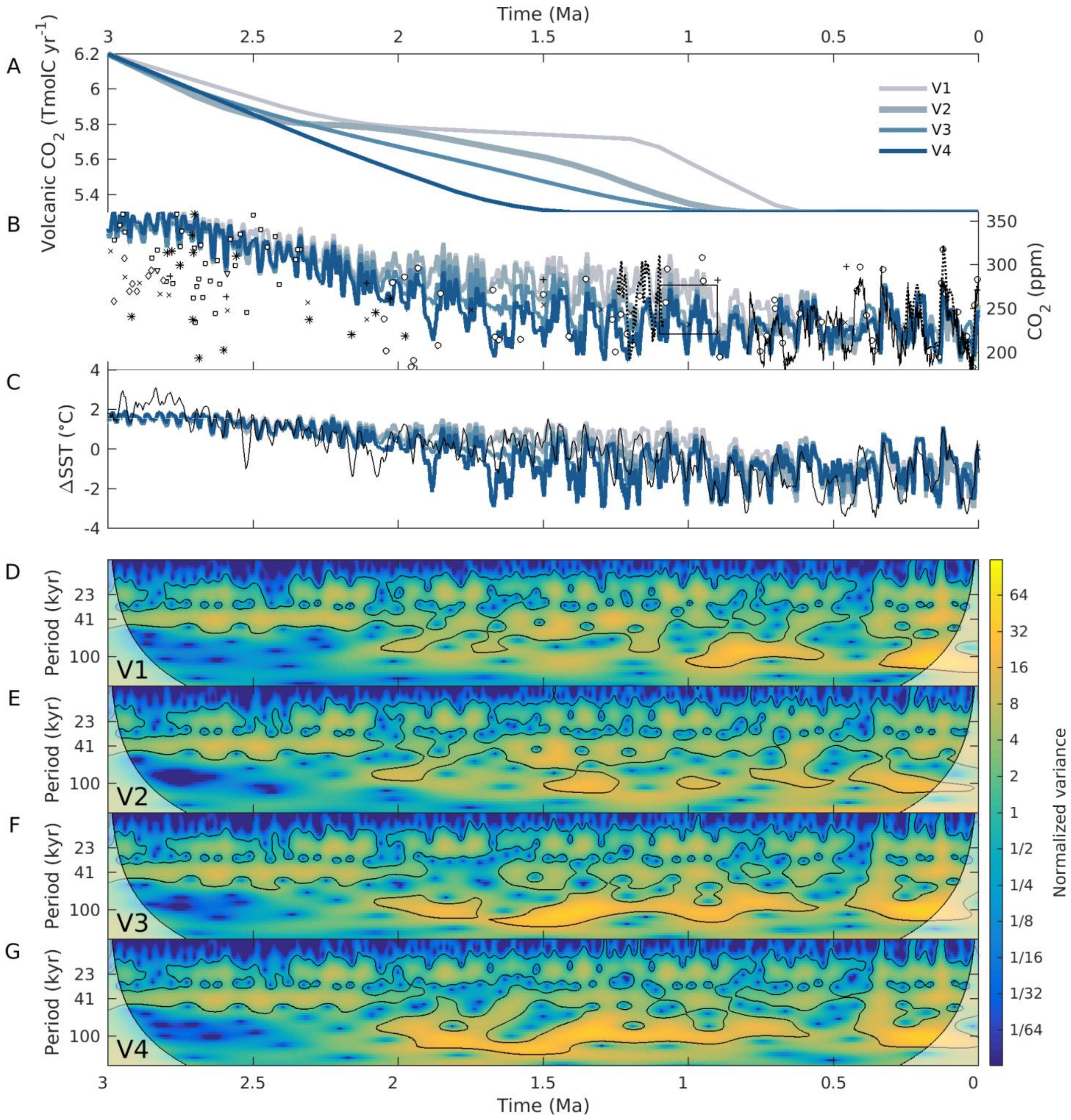

Fig. S2. Volcanic CO_2 outgassing scenarios. (**A**) Scenarios for the evolution of volcanic CO_2 outgassing and corresponding resulting modelled (**B**) atmospheric CO_2 and (**C**) sea surface temperature evolution (mean over the ensemble of simulations resulting from the time-splitting method). (**D-G**) Wavelet spectra of the modelled $\delta^{18}O$ response for simulations with volcanic CO_2 outgassing scenarios V1-V4 as indicated in the panels. Here, the model simulations are with prescribed present-day regolith cover.

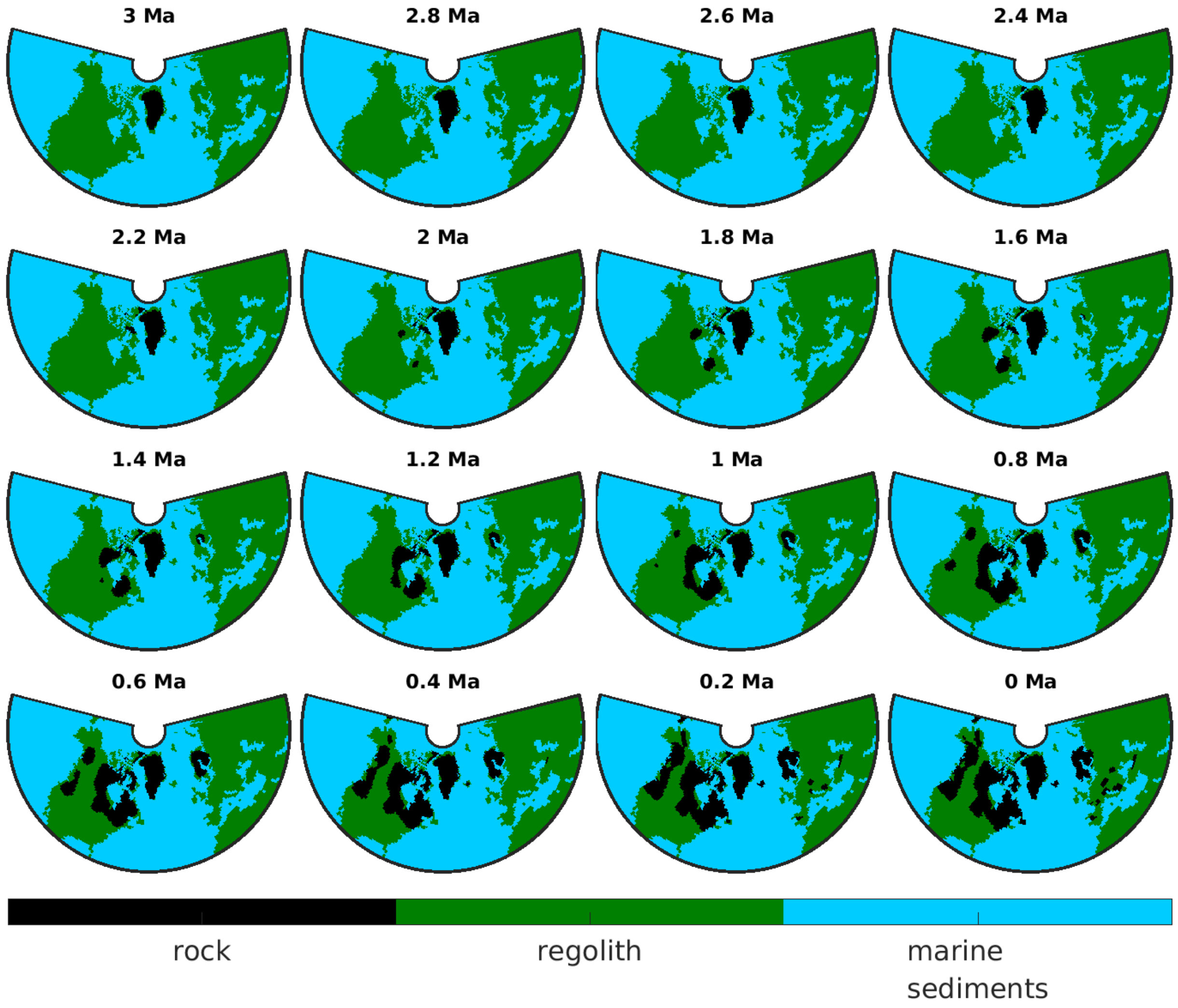

Fig. S3. Regolith removal scenario. 16 snapshots of the regolith cover mask prescribed in the model.

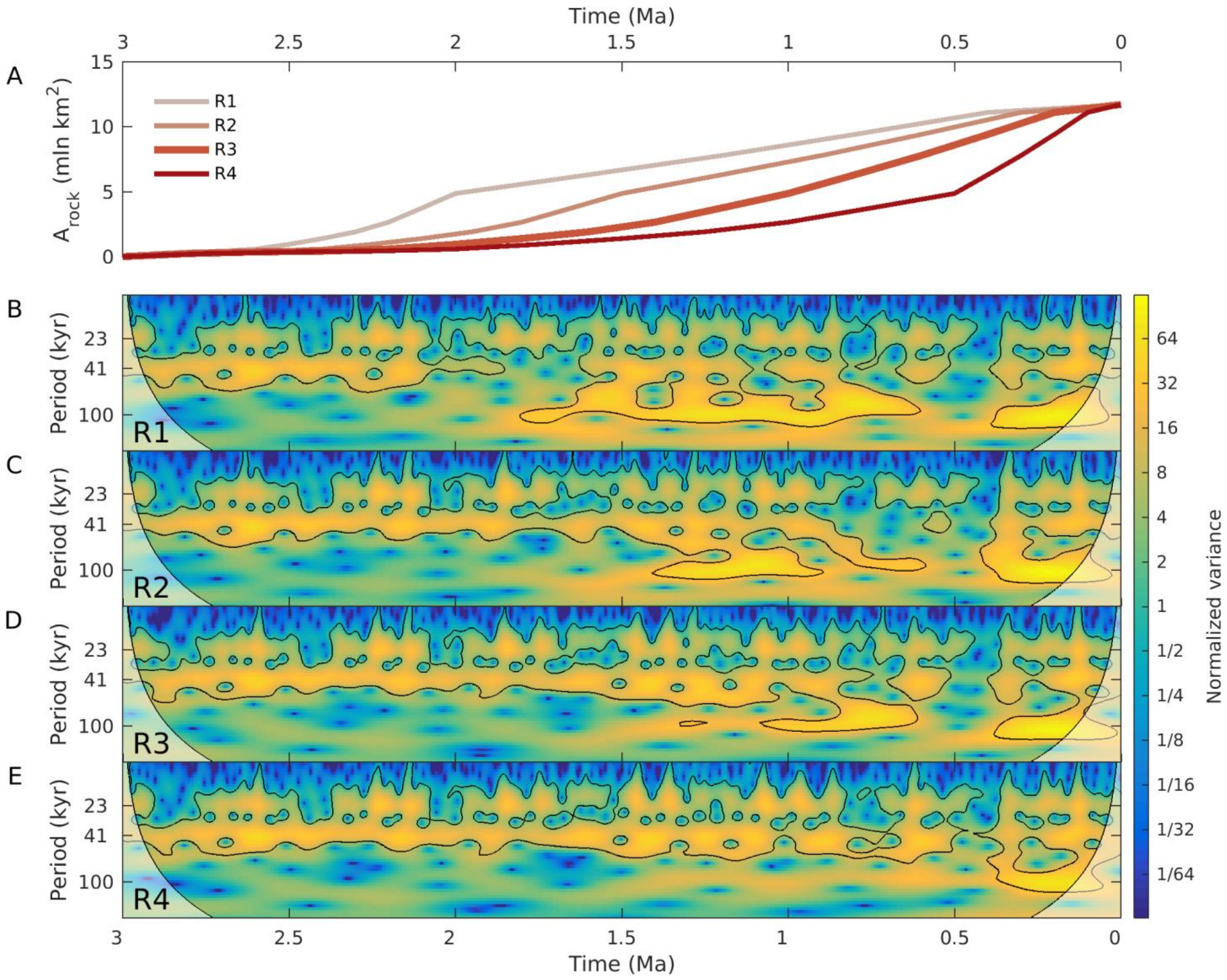

Fig. S4. Regolith scenarios. (**A**) Scenarios for the evolution of the continental (excluding Greenland) area of exposed crystalline bedrock resulting from the gradual removal of regolith by glacial erosion and (**B-E**) corresponding wavelet spectra of the modelled $\delta^{18}O$ response for simulations with the regolith scenarios R1-R4 as indicated in the panels. Here, the model simulations are with prescribed present-day volcanic CO_2 outgassing.

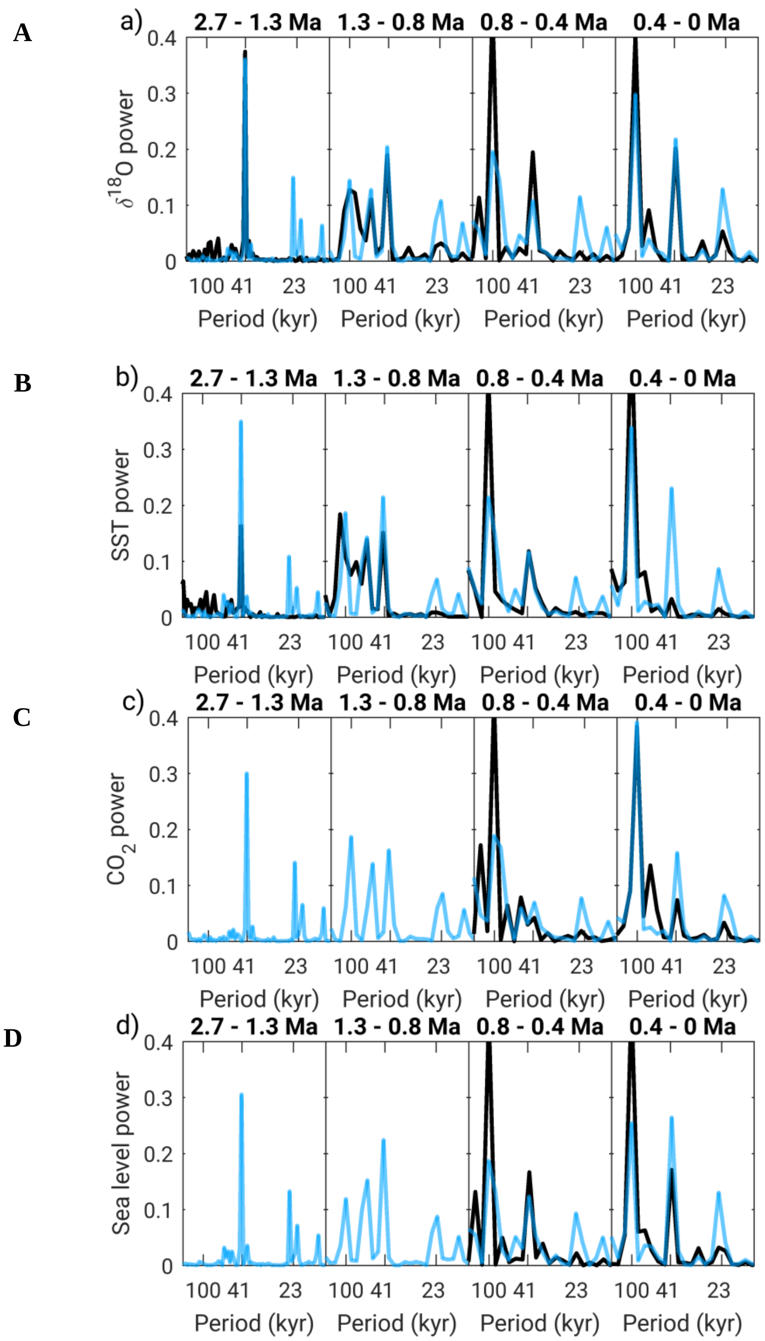

Fig. S5. Power spectra. Power spectra of (**A**) $\delta^{18}O$, (**B**) SST, (**C**) CO_2 and (**D**) sea level for different intervals of time for the simulation with optimal CO_2 outgassing and regolith removal scenarios (Fig. 1).

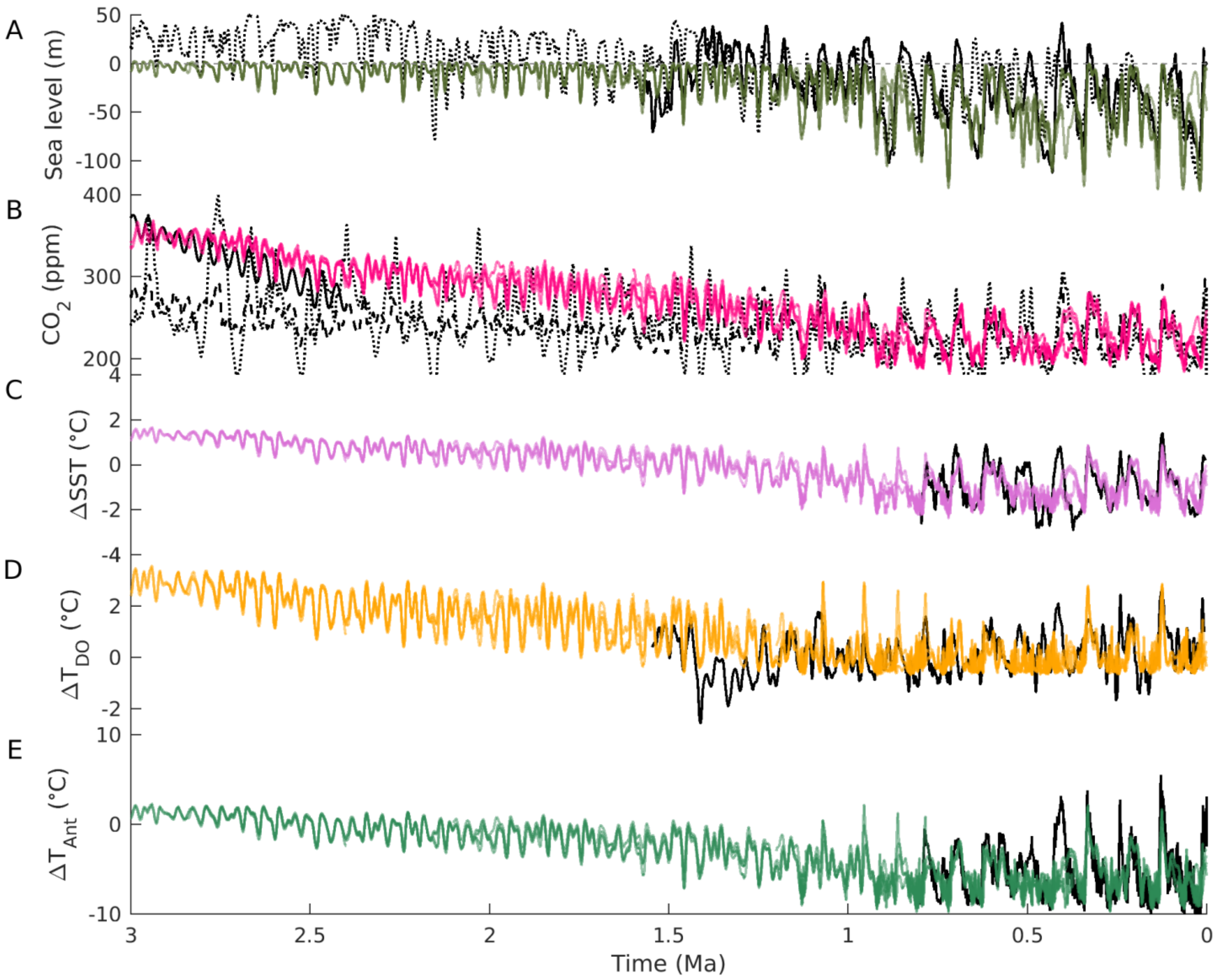

Fig. S6. Comparison to additional observations and previous modeling results. (A) Sea level compared to data from ref. (*55*) (solid black line) and ref. (*56*) (dotted black line). (B) CO_2 compared to previous modelling studies, ref. (*1*) (solid black line), ref. (*31*) (dotted black line) and ref. (*32*) (dotted black line). (C) Sea surface temperature compared to the stack of ref. (*57*). (D) Deep ocean temperature east of New Zealand compared to ref. (*55*). (E) Antarctic temperature compared to ice core proxy from ref. (*58*).

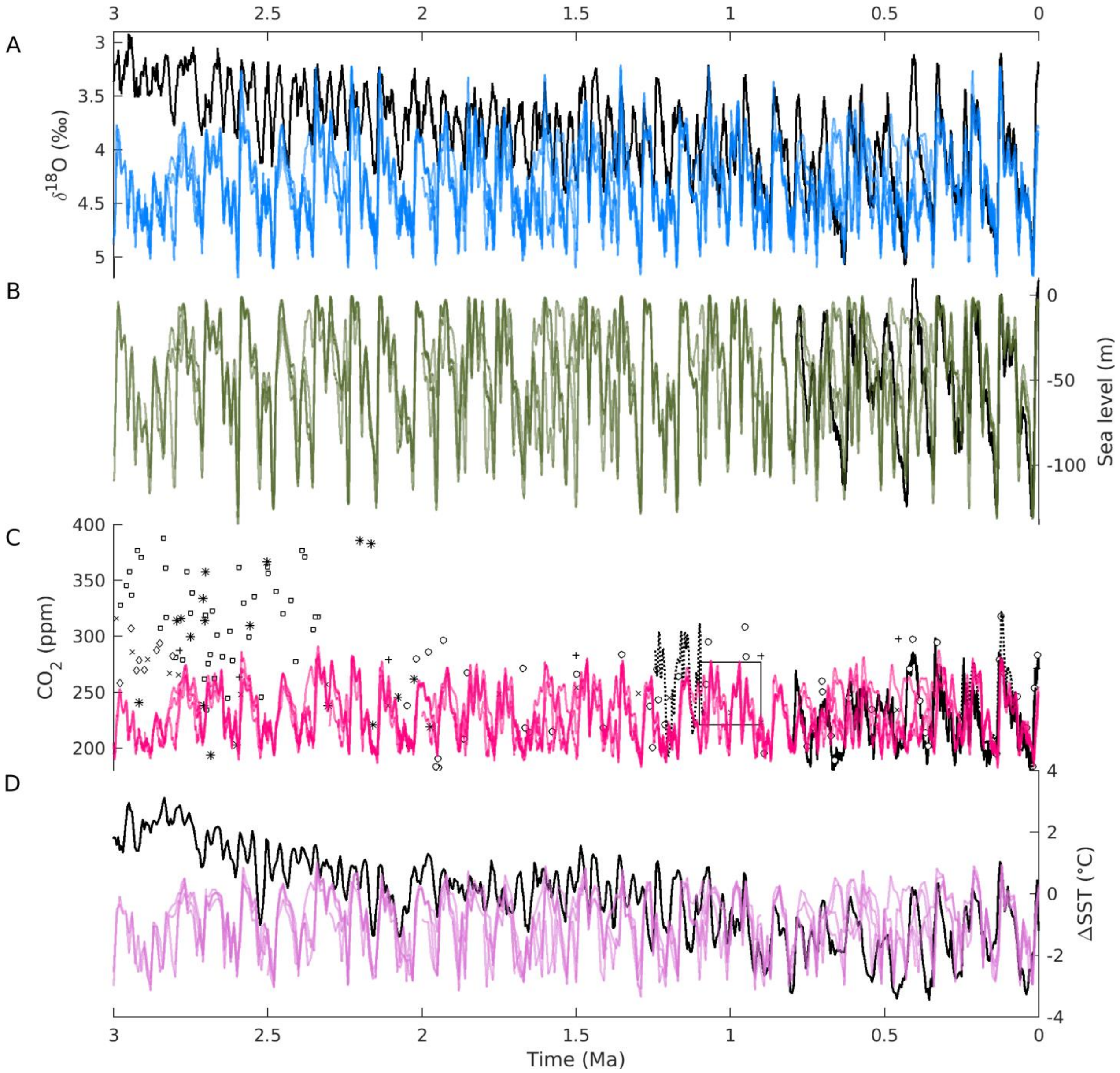

Fig. S7. Transient simulations with present-day regolith and CO_2 outgassing. Same as panels A,B,D,E in Fig. 2 but for the simulations driven only by orbital forcing with prescribed present-day regolith mask and volcanic CO_2 outgassing.

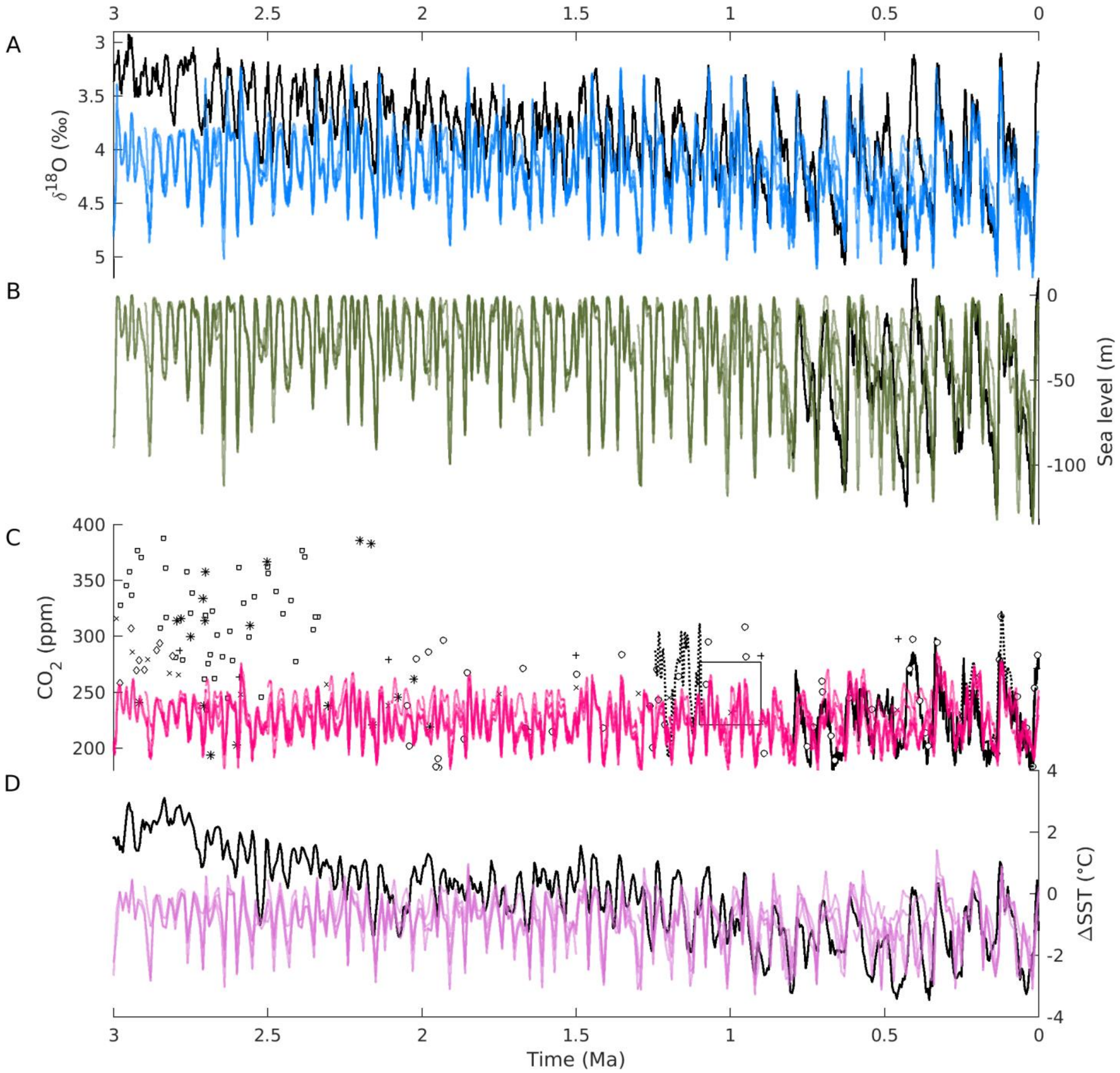

Fig. S8. Transient simulations with present-day CO_2 outgassing. Same as panels A,B,D,E in Fig. 2 but for the simulations driven only by orbital forcing and optimal regolith removal scenario with prescribed present day volcanic CO_2 outgassing.

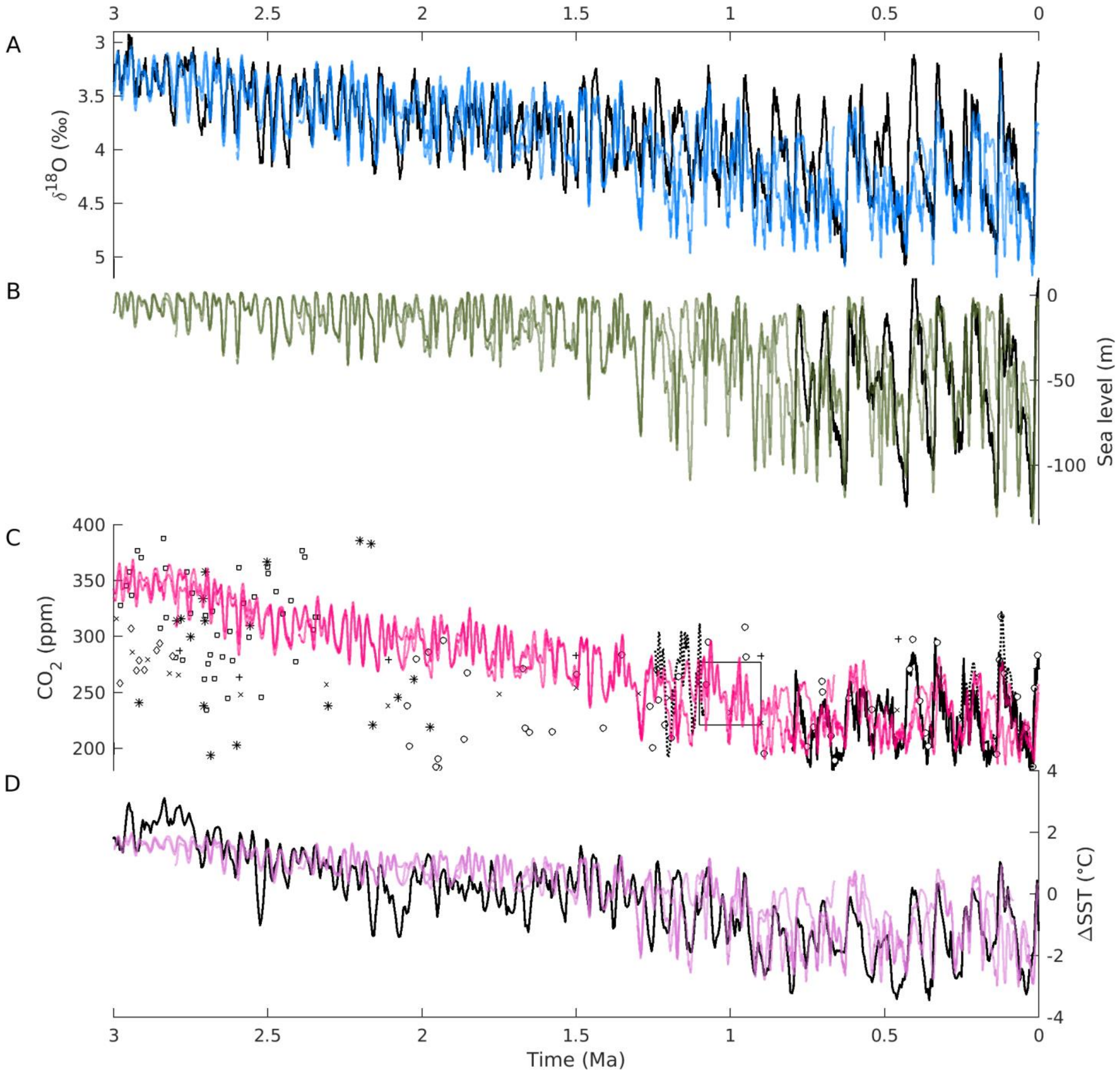

Fig. S9. Transient simulations with present-day regolith. Same as panels A,B,D,E in Fig. 2 but for the simulations driven only by orbital forcing and optimal volcanic CO_2 outgassing scenario with prescribed present day regolith mask.
